# Correlation of Internal Echo Findings With the Aggressiveness of Diffuse Large B-Cell Lymphomas: A Case Report

**DOI:** 10.7759/cureus.90626

**Published:** 2025-08-20

**Authors:** Atsuki Koyama, Shoji Oura

**Affiliations:** 1 Department of Surgery, Kishiwada Tokushukai Hospital, Kishiwada, JPN

**Keywords:** diffuse large b-cell lymphoma, dlbcl, edematous background, internal high echoes, less aggressive biology

## Abstract

It is well known that diffuse large B-cell lymphomas (DLBCLs) generally have very low internal echoes. We herein report a case of DLBCL presenting with internal high echoes and found that internal high echoes correlate with the less aggressive form of the tumor. A 68-year-old man with a history of sigmoid colon cancer and metastatic liver tumor surgeries was referred to our hospital due to multiple swollen lymph nodes, especially around the abdominal aorta. Positron emission tomography/computed tomography of the target foci showed a maximum standardized uptake value of 23. The soluble interleukin-2 receptor was elevated up to 2763 U/mL. Ultrasound showed that the masses predominantly had internal low echoes; however, there were some masses with punctate internal high echoes in the upper abdomen and these seemed most amenable to surgical removal. The patient, therefore, underwent an excisional biopsy on one of these target nodes. Pathological study of the removed node showed medium-to-large atypical cells diffusely proliferating against mixed sclerotic and edematous backgrounds. In addition, the mass had heterogeneous dense and sparse areas of atypical cells. Immunostaining of the atypical cells showed CD20, CD79a, CD5, and BCL2 positivity, CD10, CD15, CD23, CD30, BCL6, and cyclin D1 negativity, and a Ki-67 labelling index of 70%, leading to the diagnosis of DLBCL. Diagnostic physicians should note that DLBCLs can have internal high echoes when having sparse lymphoma cells against the edematous background.

## Introduction

Malignant lymphomas overwhelmingly occur in lymph nodes, and therefore generally appear as round or oval masses with clear borders on all types of image modalities. Malignant lymphomas basically have solid internal structures, but can exhibit various image findings depending on the tumor cell density and the coexisting pathological components such as adipocytes [1], fibrous components, and edematous backgrounds [2].

In addition to necks, groins, and axillae, malignant lymphomas can also develop both in the thorax [3] and the abdomen [4]. Physicians can easily make a pathological diagnosis of malignant lymphoma when it is located superficially, by directly obtaining samples from the target tissue using core needle biopsy or other techniques. Image diagnosis of the target lesion, however, becomes more important for diagnostic physicians on deciding whether to perform tissue sampling or not when finding the target tissue located deep in the thorax or abdomen.

Image diagnostic procedures are essentially the same for both intra-abdominal and intra-thoracic lesions. Despite the limited efficacy for intra-thoracic lesions, diagnostic physicians can often use ultrasound, which is noninvasive and inexpensive, for the image diagnosis of intra-abdominal lesions. It is well known that diffuse large B-cell lymphomas (DLBCLs) generally have very low internal echoes due to their aggressive proliferating characteristics, i.e., extremely similar acoustic impedance among DLBCL cells [3]. Conversely, DLBCLs with internal high echoes are extremely rare and may have different biology from that of typical DLBCLs.

We herein report a case of DLBCL with internal punctate high echoes and clarify the correlation between its internal echo findings and the aggressiveness of the DLBCL.

## Case presentation

**Figure 1:**
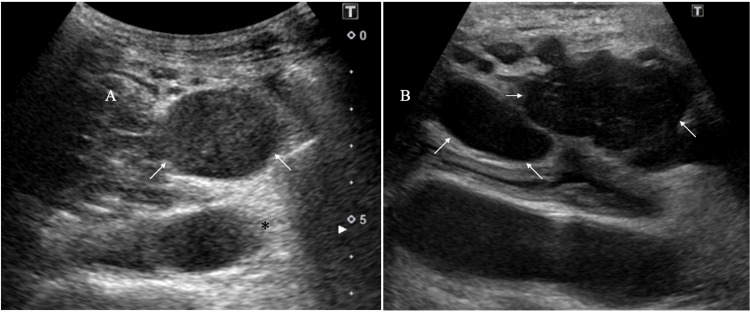
Ultrasound findings A. The target lesion (arrows) show an oval shape with clear margins, internal punctate high echoes, and enhanced posterior echoes (asterisk) B. Positron emission tomography-positive lesions (arrows) show oval or lobulated masses with very low internal echoes.

A 68-year-old man underwent surgery for moderately differentiated sigmoid colon cancer without adjuvant chemotherapy due to the patient’s preference. Two years after the operation, the patient further underwent a hepatic surgery for the liver metastasis of colon cancer, followed by eight cycles of adjuvant oxaliplatin-containing chemotherapy, and thereafter completed the five-year follow-up without any additional recurrences. Screening ultrasound, done six years after the completion of colon cancer follow-up, revealed multiple swollen lymph nodes, especially around the abdominal aorta (Figure 1).

**Figure 2:**
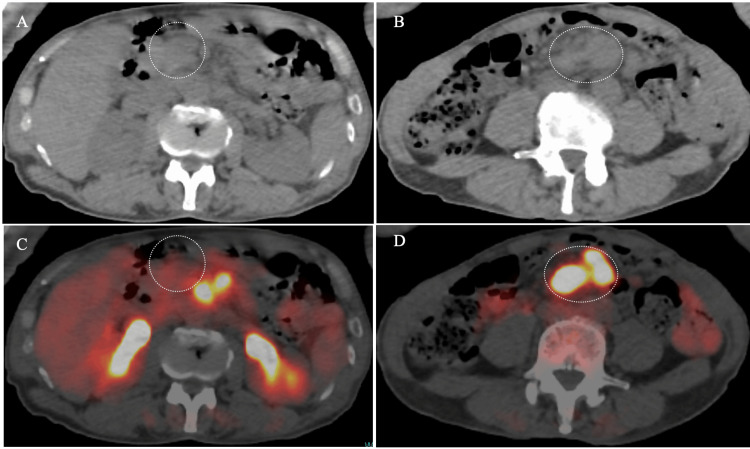
Computed tomography (CT) and positron emission tomography (PET)/CT findings (A, B): CT shows multiple swollen lymph nodes (dotted circles); PET/ CT shows no fluorodeoxyglucose uptake in the target nodes very close to the abdominal wall (C, dotted circle) and avid uptake in the lesions around the abdominal aorta (D, dotted circle).

Positron emission tomography/computed tomography (PET/CT) of the target foci showed a maximum standardized value of 23 (Lugano classification stage II) (Figure 2).

Although no increase was observed in various colorectal cancer markers, including carcinoembryonic antigen (CEA) and CA19-9, the soluble interleukin-2 receptor (sIL-2R) was elevated up to 2763 U/mL (reference range: 122-496U/mL). Ultrasound showed multiple oval or lobulated masses predominantly located around the abdominal aorta. Internal echoes of them were basically low, but those in the upper abdominal lesions, the farthest masses from the sigmoid colon cancer operation site, had internal punctate high echoes against the background low echoes.

**Figure 3:**
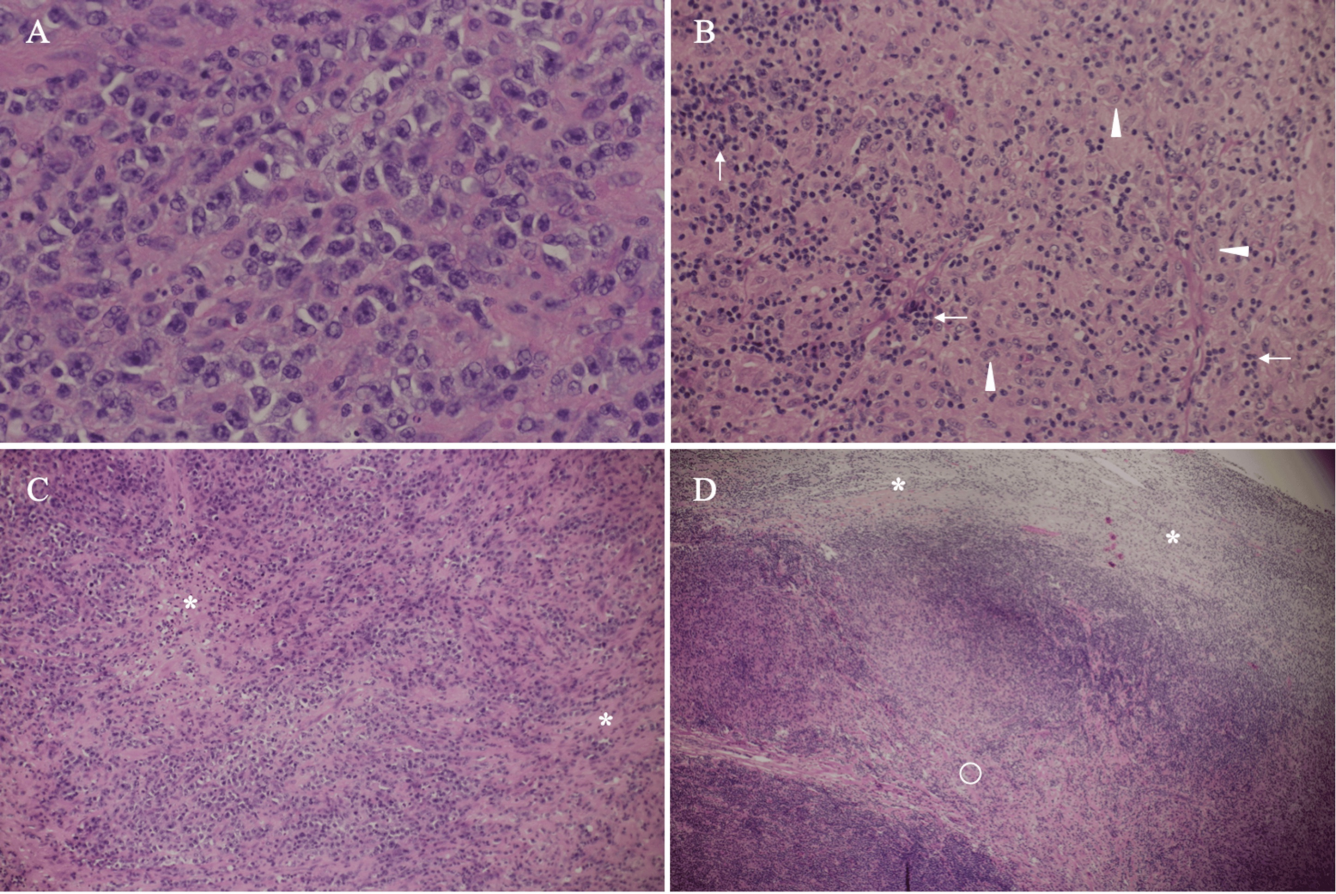
Pathological findings (A) Magnified view show tumor cells growing densely, although the cell density is somewhat low as a diffuse large B-cell lymphoma (H&E, ×200) (B) Interminglement of marked lymphocytes (arrows) and atypical cells (arrowheads) seen (H&E, ×100) (C) Fibrous components (asterisks) seen among slightly less densely packed lymphoma cells (H&E, ×40) (D) Low magnified view shows sparse lymphoma cell areas either with edematous (asterisks) or sclerotic (open circle) backgrounds in the mass (H&E, ×20)

The need for a definitive diagnosis forced us to attempt some kind of biopsy on at least one target lesion. We, therefore, decided to biopsy the upper abdominal masses with internal high echoes as the target nodes, i.e., seemed amenable to surgical removal most easily, followed by further biopsy for deeper masses if the initial frozen section could not provide meaningful pathological findings. In the biopsy operation, the ultimate target lesion, which we aimed to resect, was an enlarged and fused lymph node within the small intestinal mesentery and was easily resected. Frozen section showed malignant lymphoma cells and therefore made us not resect more lesions. Postoperative pathological study showed medium-to-large atypical cells diffusely proliferating against the mixed sclerotic and edematous backgrounds (Figure 3).

**Figure 4:**
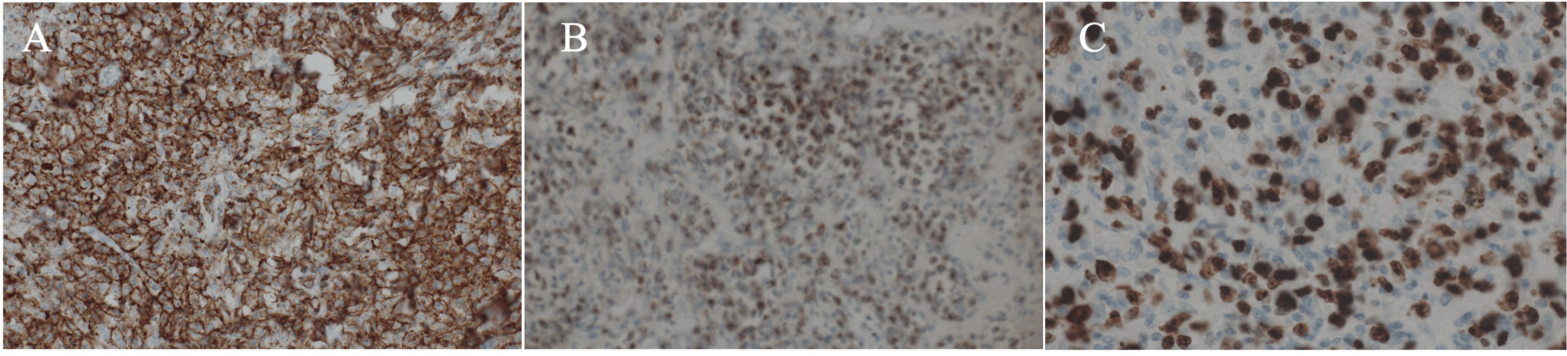
Immunostaining findings Tumor cells positive both for CD20 (A) and BCL2 (B), with a very high Ki-67 labelling index of 70%.

In addition, the mass had heterogeneous areas of sparse and relatively dense lymphoma cells. Immunostaining of the DLBCL cells showed CD20, CD79a, CD5, and BCL2 positivity, CD10, CD15, CD23, CD30, BCL6, and cyclin D1 negativity, and a Ki-67 labelling index of 70%, leading to the diagnosis of DLBCL (Figure 4).

The patient recovered uneventfully, was discharged on the third day after surgery, and was referred to another hospital for chemotherapy.

## Discussion

Reflection and backscattering of ultrasound waves generate shapes and internal echoes of masses, respectively [5]. Ultrasound waves reflect at the interfaces, much larger than the ultrasound beam width, while they backscatter at much smaller scattering bodies. Intensity of reflection and backscattering is determined by the difference of acoustic impedance between interfaces/scattering bodies and the surrounding pathological components. In other words, the echogenicity of tumor margins or its internal areas increases as the difference in acoustic impedances of adjacent pathological components increases [6].

This patient underwent surgery for moderately differentiated, i.e., a cribriform dominant phenotype, adenocarcinoma of the sigmoid colon 11 years before. It is well known that breast cancers with cribriform and tubular structures have internal high echoes due to the difference in acoustic impedance between cancer cells and their surrounding microvoids in these structures [7]. It is also well known that elevation of sIL-2R levels suggests T cell activation, found not only in malignant lymphomas but also in various disorders, including sarcoidosis [8]. We, therefore, also took the possible colon cancer recurrence with a very long disease-free interval into consideration before performing the biopsy.

DLBCLs are highly aggressive disorders and generally form masses pathologically consisting of very similar atypical cells. Therefore, the least backscattering of ultrasound waves often generates very low internal echoes in DLBCLs due to little difference in acoustic impedance among lymphoma cells [9]. The target mass, however, had abundant fibrous components, which are scarcely observed in the vast majority of DLBCL lesions. In addition, we have already shown that tumor cells and lymphocytes present against edematous backgrounds can cause ultrasound wave backscattering, leading to internal high echo formation [4]. These facts, therefore, strongly suggest that punctate high echoes in the mass were caused by lower intra-tumoral cell density than that in typical DLBCLs.

Pre-biopsy PET images showed no fludeoxyglucose (FDG) uptake in the target lesion. This finding is also well explained by the lower tumor cell density, abundant presence of fibrous components, and the edematous background. It is naturally unknown what kind of pathological findings the PET-positive nodes had because we did not resect them. It is, however, not difficult for us to imagine that they likely had similar pathological findings to those of typical DLBCLs, given that the internal echoes of them were very low.

This study has the limitation of being based on the pathological evaluation of a single biopsied lymph node. Furthermore, many lymph nodes, not biopsied, had very low internal echoes in this case. It is, therefore, naturally unclear how the interminglement of less aggressive foci affects clinical outcomes of DLBCL lesions. It, however, is very important for physicians to be aware that DLBCLs with these atypical images exist.

It is extremely important for diagnostic physicians to understand the typical images of various disorders, not just DLBCLs. However, the presence of various pathological components such as fibrous components, edema, fat, and mucus within the tumor can generate images that are markedly different from typical DLBCL images [6]. It is, therefore, very important for us to know how each pathological component affects various image modalities for correct image diagnosis.

## Conclusions

This study was based on a single case and naturally needs further investigation. Diagnostic physicians, however, should note that DLBCLs with internal high echoes can have less aggressive pathological components such as sparse lymphoma cells, fibrous components, and the edematous background. In other words, physicians should keep in mind that there is a close correlation between the internal echo findings of DLBCLs and their biology when diagnosing and treating them.
